# Electroacupuncture Attenuates Fibromyalgia Pain via Toll-like Receptor 4 in the Mouse Brain

**DOI:** 10.3390/life13051160

**Published:** 2023-05-11

**Authors:** Po-Chih Lai, Chia-Ming Yen, Ming-Chia Lin, Yung-Hsiang Chen, Hsien-Yin Liao, Yu-Wei Huang, Yi-Wen Lin

**Affiliations:** 1 College of Chinese Medicine, Graduate Institute of Acupuncture Science, China Medical University, Taichung 40402, Taiwan; 2 Department of Anesthesiology, Taichung Tzu Chi Hospital, Buddhist Tzu Chi Medical Foundation, Taichung 42743, Taiwan; 3 School of Post-Baccalaureate Chinese Medicine, Tzu Chi University, Hualien 97004, Taiwan; 4 Department of Nuclear Medicine, E-DA Hospital, College of Medicine, I-Shou University, Kaohsiung 82445, Taiwan; 5 Graduate Institute of Integrated Medicine, China Medical University, Taichung 40402, Taiwan; 6 Department of Psychology, College of Medical and Health Science, Asia University, Taichung 41305, Taiwan; 7 College of Chinese Medicine, School of Post-Baccalaureate Chinese Medicine, China Medical University, Taichung 40402, Taiwan; 8 Emergency and Critical Care Center, E-Da Hospital, Kaohsiung 80708, Taiwan; 9 Department of Nursing, School of Nursing, Fooyin University, Kaohsiung 83102, Taiwan; 10 School of Medicine, College of Medicine, I-Shou University, Kaohsiung 824, Taiwan; 11 Chinese Medicine Research Center, China Medical University, Taichung 40402, Taiwan

**Keywords:** electroacupuncture, fibromyalgia, hypothalamus, microglia, TLR4, cerebellum

## Abstract

Background: Fibromyalgia (FM) is characterized by complex pain symptoms lacking impersonal considerations in diagnosis and treatment evaluation, which often happens in women. Chronic and persistent widespread pain is the key symptom disturbing patients with FM, leading to depression, obesity, and sleep disturbances. Toll-like receptor 4 (TLR4) activation produces a harmful sensory input involved in central pain; this is the focus of this study. Electroacupuncture (EA) has beneficial effects in reducing FM pain, but its connection with TLR4 signaling is still unknown. Methods: Intermittent cold stress significantly induced mechanical and thermal pain. EA, but not sham EA, reliably attenuated mechanical and thermal hyperalgesia. The increased inflammatory mediators in FM mice were reduced in the EA group, but not in the sham group. Results: All TLR4 and related molecule levels increased in the FM mice’s hypothalamus, periaqueductal gray (PAG), and cerebellum. These increases could be attenuated by EA but not sham stimulation. Activation of TLR4 by lipopolysaccharide (LPS) significantly induced FM and can be further reversed by a TLR4 antagonist. Conclusions: These mechanisms provide evidence that the analgesic effect of EA is related to the TLR4 pathway. In addition, we showed that inflammation can activate the TLR4 pathway and provided new possible therapeutic targets for FM pain.

## 1. Introduction

Fibromyalgia (FM) is accompanied by chronic general musculoskeletal pain lasting over 3 months and is highly associated with sleep disturbances, fatigue, and cognitive and somatic symptoms. These symptoms may persist over several years and cause extensive healthcare use [1]. For clinical diagnosis, FM can be characterized by trigger pain with tenderness in 18 special points [2]. The prevalence of FM ranges from 0.2 to 4.7%; almost 90% of the cases occur in women, of which >50% were aged 40–60 years. The new FM diagnosis is based on widespread pain index (WPI) ≥ 7 and a symptom severity scale (SS) ≥ 5 or WPI 3–6 and SS ≥ 9 for over 3 months [3,4]. The standard treatment for FM is a combination of pharmacological and behavioral treatments [4,5]. The most used FM drugs are antidepressants (serotonin–norepinephrine reuptake inhibitors, SNRI) and GABAergic medications (pregabalin). Furthermore, exercise, meditation, cognitive behavioral treatments, and integrative medicine, including acupuncture, were reported to cause pronounced improvements in FM [6,7,8]. Several FM animal models, including acidic saline injection [9], sound stress [10], and cold stress [11,12], have been used to mimic FM pain.

Toll-like receptor (TLR) 4 is a type I transmembrane protein that can be activated by bacterial lipopolysaccharide (LPS), further producing proinflammatory cytokines and interferons to initiate inflammatory and immune responses [13,14]. TLRs can be sub-divided based on cell location. TLR1, TLR2, TLR4, TLR5, and TLR6 are found on the cell membrane. In addition, TLR3, TLR7, TLR8, and TLR9 are located inside endosomes [15]. TLR4 can be activated by damage-associated molecules that were released from damaged or dying cells. In neuroinflammation, the innate immune system, including the microglia or astrocytes, can be activated by inflammatory mediators. The activated TLR4 then initiated intracellular signals on two distinct pathways such as MyD88/NF-κB and TRIF/IRF3 [14]. The TLR4 signaling pathway has been involved in major depressive disorders [16] and peripheral neuropathic pain [17]. A loss-of-function mutation in the Tlr4 gene reliably reduced microglial activation, decreasing the concentration of inflammatory mediators [18]. TLR4 antagonism attenuated NF-κB pathways to decrease the number of inflammatory mediators secreted by astrocytes [19]. Furthermore, TLR4 plays a crucial role in central neuroinflammation, including IL-1β upregulation [20].

Acupuncture has long been used as an integrative therapy for several diseases, including pain management, with a lower risk of side effects. Traditional manual acupuncture and electroacupuncture (EA) have been reported to result in pain improvement. Acupuncture has been reported to attenuate inflammatory pain [21], osteoarthritis [22], neuropathic pain [23], and FM pain in mice [9,12]. In EA, fine steel needles are inserted into specific points to deliver a series of electrical stimulations. The real mechanisms of EA are evidence-based but still a pending mystery. Recent research proposes that EA can initiate the release of endogenous opioids [24], dopamine [25], and adenosine [26] to reduce the symptoms. Furthermore, EA has also been shown to reduce FM pain via the downregulation of inflammatory factors such as interleukins, TNF-α, and IFN-γ in mouse plasma [12]. Moreover, Liu et al. reported that acupuncture could significantly drive the vagal-to-adrenal instead of sympathetic reflexes [27].

The current study aimed to explore the effect of EA on cold-stress-induced FM pain (CSP). Particularly, we wanted to identify whether inflammatory mediators and related signaling pathways were involved in EA analgesia in the mouse brain. We hypothesized that inflammatory mediators would increase in CSP mice and activate various receptors, including TLR4. Our data show new evidence of EA and TLR4 in the central sensitization of CSP. Furthermore, we propose that EA can attenuate CSP by regulating inflammatory factors and related molecules. Activation of TLR4 by LPS significantly induced CSP and can be further reversed by a TLR4 antagonist. These results may suggest the clinical applicability of EA for treating CSP.

## 2. Materials and Methods

### 2.1. Animals

A total of 40 female C57BL/6 mice, which were aged 8–12 weeks old, were used in this study. After their arrival, the animals were held in a 12 h light–dark cycle and received food and water freely. The anticipated model size of 10 mice per group was counted as the amount needed for an alpha value of 0.05 and a power of 80%. The use of mice and their suffering were reduced according to animal research ethical standards. The usage of mice was allowed by the Institute of Animal Care and Use Committee of China Medical University (grant no. CMUIACUC-2019-106), Taiwan, and followed the Guide for the Use of Laboratory Animals (National Academy Press, Washington, DC, USA). Mice were allocated into four main groups: normal mice (Group 1: normal); fibromyalgia mice (Group 2: FM); 2 Hz EA group (Group 3: 2 Hz EA); and sham EA group (Group 4: sham EA).

### 2.2. FM Model and Bio-Plex ELISA

Mice were maintained in the laboratory at 24 ± 1 °C before the experiments. The intermittent FM process, which was not achieved in normal mice, required that two mice were accommodated in a flexible cage (13 cm × 18.8 cm × 29.5 cm) that was circumscribed with a stainless mesh. On day 0, the mice were placed in a cold chamber at 4 °C overnight (from 4:00 p.m. to 10:00 a.m.). The mice were subsequently moved to 24 °C for 30 min at 10:00 a.m. and further transported to the cold room for 30 min. The course continued till 4:00 p.m. The mice were then placed in the 4 °C cold room overnight. Normal mice were maintained at 24 ± 1 °C [12]. Mouse plasma was collected and confirmed through Q-view cytokine assays (Q-view, Los Altos, CA, USA).

### 2.3. EA Treatments

All mice were anesthetized with 5% isoflurane at the beginning and 1% isoflurane for maintenance. Stainless-steel acupuncture needles (1 inch, 36 G, YU KUANG, Taipei, Taiwan) were bilaterally implanted into the ST36 acupoints of mice under anesthesia. The mouse ST36 acupoint is located ~3–4 mm deep and 1–2 mm lateral to the knee joint. In the EA group, electrical stimuli were delivered by a Trio 300 stimulator (Ito, Tokyo, Japan) by using 1 mA for 20 min at 2 Hz with a pulse width of 100 μs. Muscle twitching was observed during EA treatment as a de-qi sensation. Subsequent FM induction, EA management was performed from day 5 to 7. The same protocol was applied in the sham group but without electrical stimulation [12].

### 2.4. Pain Behavior Test

After FM induction, either mechanical or thermal hyperalgesia was verified thrice from day 5 to day 7. Before behavior examinations, mice were relocated to the behavioral room and left to adjust to the room for ≥30 min. All tests were achieved at 24 ± 1 °C; the stimuli were tested when the mice were silent. Mechanical hyperalgesia was confirmed by applying force to stimulate trice the electronic von Frey filament (IITC Life Science Inc., Woodland Hills, CA, USA). Furthermore, the Hargreaves’ test was applied to measure thermal hyperalgesia by analyzing the latency-to-thermal stimulation thrice by using Hargreaves’ test IITC analgesiometer (IITC Life Sciences, SERIES8, Model 390G). In the Hargreaves’ test, the pain threshold was measured via the latency of paw withdrawal time [12].

### 2.5. Western Blotting Analysis

Mice were anesthetized with 1% isoflurane and then underwent cervical dislocation. The hypothalamus, periaqueductal gray (PAG), and cerebellum VI and VII sections were removed for protein extraction. Samples were first stored at 4 °C and then kept at −80 °C, awaiting protein extraction. Sample proteins were homogenized with a radioimmunoprecipitation lysis buffer containing 50 mM Tris–HCl pH 7.4, 250 mM NaCl, 1% NP-40, 0.5% SDS, 0.5% deoxycholate, 5 mM EDTA, 50 mM NaF, 1 mM Na_3_VO_4_, 0.02% NaN_3_, and 1× protease inhibitor cocktail (AMRESCO, Solon, OH, USA). The proteins were further exposed to 8% Tris-Glycine-SDS glycine gel electrophoresis and transferred to a polyvinylidene difluoride membrane. The membrane was blocked with 5% nonfat milk in TBS-T buffer (10-mM Tris pH 7.5, 100-mM NaCl, and 0.1% Tween 20) and incubated with a primary antibody against anti-tubulin (∼55 kDa, Merck, Rahway, NJ, USA; cat. no. 05-829; 1:5000), anti-Iba1 (∼15 kDa, Alomone, Israel; cat. no. ACS-010; 1: 1000), anti-TLR4 (∼35 kDa, Merck, USA; cat. no. AP1179; 1:1000), anti-MyD88 (~35 kDa, Cell signaling, Danvers, MA, USA; cat. no. 4283; 1:1000), anti-TRAF6 (∼60 kDa, Cell signaling, USA; cat. no. 8028; 1:1000), anti-pERK (~42–44 kDa, Cell signaling, USA; cat. no. 4695; 1:1000), anti-pJNK (∼60 kDa, Thermo Fisher Scientific, Waltham, MA, USA; cat. no. 44-682G; 1:1000), anti-pp38 (∼42 kDa, Thermo Fisher Scientific, USA; cat. no. 44-684G; 1:1000), and anti-pNFκB (Merck, USA; cat. no. ABS403; 1:1000) in TBS-T with 1% bovine serum albumin (BSA) for 1 h at 24 ± 1 °C. A commercial peroxidase-conjugated anti-rabbit antibody, anti-mouse antibody, or anti-goat antibody (1:5000) were used as secondary antibodies. Western blot bands were imagined through a heightened chemiluminescent substrate kit (PIERCE, Waltham, MA, USA) with LAS-3000 Fujifilm (Fuji Photo Film Co., Tokyo, Japan, Ltd.). The concentration of each band was quantified by using NIH Image J 1.46 software (Bethesda, MD, USA). α-tubulin was set as the internal controller.

### 2.6. Immunofluorescence

Mice were anesthetized with 5% isoflurane and intracardially perfused with 0.9% saline and then 4% paraformaldehyde. The mouse brain was proximately separated and postfixed by 4% paraformaldehyde at 4 °C for over 3 days. The brain was then immediately stored in 30% sucrose for cryoprotection for 24 h at 4 °C. Then, the sample was quickly frozen by using liquid nitrogen and then stored at −80 °C. Frozen sections (12 images captured per group) were cut at 20 μm thickness and immediately placed on glass slides. The tissues were then fixed with 4% paraformaldehyde and incubated with a blocking solution (3% BSA, 0.1% Triton X-100, and 0.02% sodium azide) for 1 h at 24 ± 1 °C. The tissues were then incubated with the primary antibody against anti-Iba1 (Alomone, Jerusalem, Israel; cat. no. ACS-010; 1:200; Polyclonal), anti-TLR4 (Merck, USA; cat. no. AP1179; 1: 200; monoclonal) in 1% BSA solution at 4 °C overnight. Tissues were further incubated with secondary antibodies (1: 500), 488-conjugated AffiniPure donkey anti-rabbit IgG (H + L), 594-conjugated AffiniPure donkey anti-goat IgG (H + L), and peroxidase-conjugated AffiniPure donkey anti-mouse IgG (H + L) for 2 h at 24 ± 1 °C. Tissues were detected by using an epifluorescence microscope (Olympus, BX-51, Shinjuku City, Japan) with a 20× numerical aperture (NA = 1.4) objective. Image colocalization was adjusted by using NIH Image J 1.46 software.

### 2.7. Intracerebroventricular Injection

Mice were anesthetized by using isoflurane with their heads fixed in a stereotaxic cannula, and the cannula was implanted at the ventricle site. The stereotaxic cannula was subsequently placed 0.5 mm in the anteroposterior axis, ±1 mm in the mediolateral axis, and −2.5 mm in the dorsoventral axis below the cortical surface. This cannula was a 23 gage, including a 2 mm stainless steel. In addition, it was immobile at the skull with a dental glue. Subsequently, the cannula was implanted and linked to a Hamilton syringe with a PE tube (PE10, Portex, Kent, UK). Totally, 5 μL of LPS/PBS (5 μL/ventricle) was injected over a period of 5 min by using a syringe pump (KD Scientific, Shanghai, China). After the injection, the cannula was left at the ventricle for an additional 2 min to allow the LPS to diffuse. A 25 μM TIRAP was used as an antagonist of TLR4.

### 2.8. Statistical Analysis

Statistical analysis was performed with SPSS statistical software. All results are shown as mean ± standard error (SEM). Shapiro–Wilk examination was used to test the normal distribution of the data. Statistical differences were investigated through repeated-measures ANOVA tests. The post hoc Tukey’s test was performed after ANOVA. *p* < 0.05 indicated the threshold for statistical significance.

## 3. Results

### 3.1. Electroacupuncture Attenuated Fibromyalgia-like Pain in Mice

**Figure 1:**
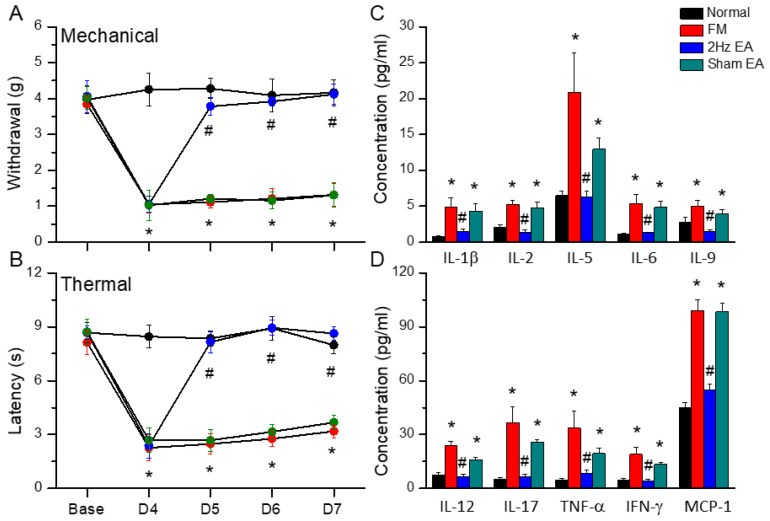
Mechanical, thermal hyperalgesia and inflammatory mediators in normal, FM, 2Hz EA, and sham EA mice. (**A**) Mechanical threshold from the von Frey tests. (**B**) Thermal latency from the Hargreaves’ test. (**C**) IL-1β, IL-2, IL-5, IL-6, and IL-9. (**D**) IL-12, IL-17, TNF-α, IFN-γ, and MCP-1. * indicates statistical significance when compared to the normal mice. # indicates statistical significance when compared to the FM groups. *n* = 10 in all groups.

We tested the therapeutic effects of EA in a mouse FM model to address EA function in the subacute pain model to verify whether EA can reduce FM. Before FM induction, all groups showed similar mechanical thresholds. Cold stress significantly induced mechanical pain (Figure 1A, red column, D7: 1.32 ± 0.31 g, * *p* < 0.05, F(3, 36) = 24.41, *n* = 10), as shown by the von Frey test. Mechanical hyperalgesia was substantially alleviated by EA, but not sham EA (Figure 1A, blue and green columns, D7: 4.12 ± 0.29 g and 1.31 ± 0.34 g, *n* = 10, respectively). This cold stress model reliably mimicked clinical symptoms, including thermal hyperalgesia. Next, we examined whether EA could alleviate thermal pain in FM mice. The Hargraves’ test showed significant thermal hyperalgesia, as measured by the paw withdrawal latency after CSP induction (Figure 1B, red column, D7: 3.18 ± 0.39 s, * *p* < 0.05, F(3, 36) = 30.02, *n* = 10). The hyperalgesic latency could be further reversed by EA but not by sham EA, suggesting an acupoint-specific effect (Figure 1B, D7: 8.63 ± 0.39 s and 3.68 ± 0.38 s, *n* = 10, respectively).

### 3.2. Electroacupuncture Treatment Alleviated the Increase in Inflammatory Cytokines Caused by Cold-Stress-Induced Pain

Since central inflammation was described in clinical patients, we next examined inflammatory mediators in FM mice via Bio-Plex ELISA. In normal mice, several inflammatory cytokines were recorded at a low level in basal conditions. Furthermore, we found that IL-1β, IL-2, IL-5, IL-6, IL-9, IL-12, IL-17A, TNF-α, IFN-γ, and MCP-1 all increased in FM mice 7 days after induction (Figure 1C,D, IL-1β: 4.85 ± 1.32 pg/mL, IL-2: 5.25 ± 0.57 pg/mL, IL-5: 20.84 ± 5.55 pg/mL, IL-6: 5.37 ± 1.3 pg/mL, IL-9: 5.04 ± 0.84 pg/mL, IL-12: 23.58 ± 2.72 pg/mL, IL-17A: 36.56 ± 8.97 pg/mL, TNF-α: 33.55 ± 9.56 pg/mL, IFN-γ: 19.02 ± 3.7 pg/mL, and MCP-1: 98.97 ± 6.1 pg/mL, * *p* < 0.05, *n* = 9, red column). Additionally, after a series of EA treatments, inflammatory cytokine levels were dramatically decreased (Figure 1C,D, IL-1β: 1.53 ± 0.31 pg/mL, IL-2: 1.39 ± 0.29 pg/mL, IL-5: 6.3 ± 0.85 pg/mL, IL-6: 1.32 ± 0.08 pg/mL, IL-9: 1.48 ± 0.27 pg/mL, IL-12: 6.66 ± 1.14 pg/mL, IL-17A: 6.29 ± 1.73 pg/mL, TNF-α: 8.32 ± 2.1 pg/mL, IFN-γ: 3.99 ± 1.08 pg/mL, and MCP-1: 54.86 ± 3.1 pg/mL, # *p* < 0.05, *n* = 10, blue column), which did not occur with sham EA treatment (Figure 1C,D, IL-1β: 4.35 ± 1.02 pg/mL, IL-2: 4.83 ± 0.7 pg/mL, IL-5: 12.95 ± 1.55 pg/mL, IL-6: 4.87 ± 0.85 pg/mL, IL-9: 3.93 ± 0.57 pg/mL, IL-12: 16.04 ± 1.26 pg/mL, IL-17A: 25.88 ± 1.3 pg/mL, TNF-α: 19.4 ± 3.04 pg/mL, IFN-γ: 13.37 ± 0.87 pg/mL, and MCP-1: 98.46 ± 4.6 pg/mL, # *p* > 0.05, *n* = 10, green column).

### 3.3. Electroacupuncture but Not Sham Electroacupuncture Attenuated Cold-Stress-Induced Pain via TRPV1 Pathways in the Mouse Hypothalamus and Periaqueductal Gray

**Figure 2:**
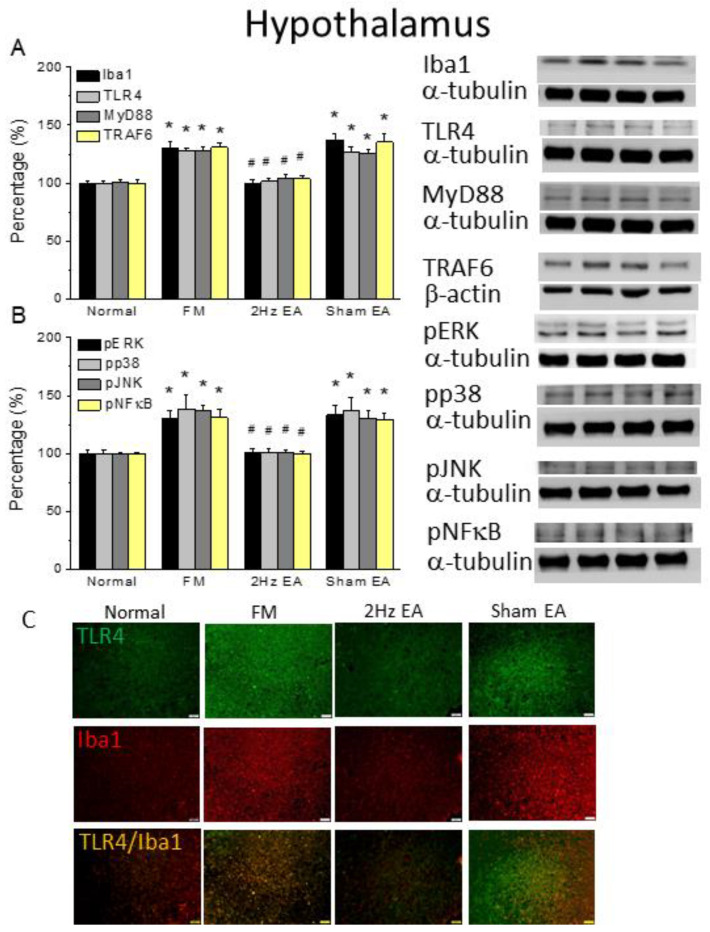
The levels of TLR4 and related molecules in the mice hypothalamus. The Western blot bands have four lanes of protein expression in the order of Normal, FM, 2Hz EA, and sham EA groups. There are significant increases in (**A**) Iba1, TLR4, MyD88, and TRAF6. (**B**) pERK, pp38, pJNK, and pNFκB protein levels in the FM group. * indicates statistical significance when compared with the normal group. # indicates statistical significance when compared to the CSP group. *n* = 6 in all groups. (**C**) Immunofluorescence staining of TLR4, Iba1, and double-staining protein expression in the mice hypothalamus. TLR4, Iba1, and TLR4/Iba1 double-staining, immuno-positive (green, red, or yellow) signals in the mice hypothalamus region. Scale bar means 100 μm. *n* = 4 in all groups.

**Figure 3:**
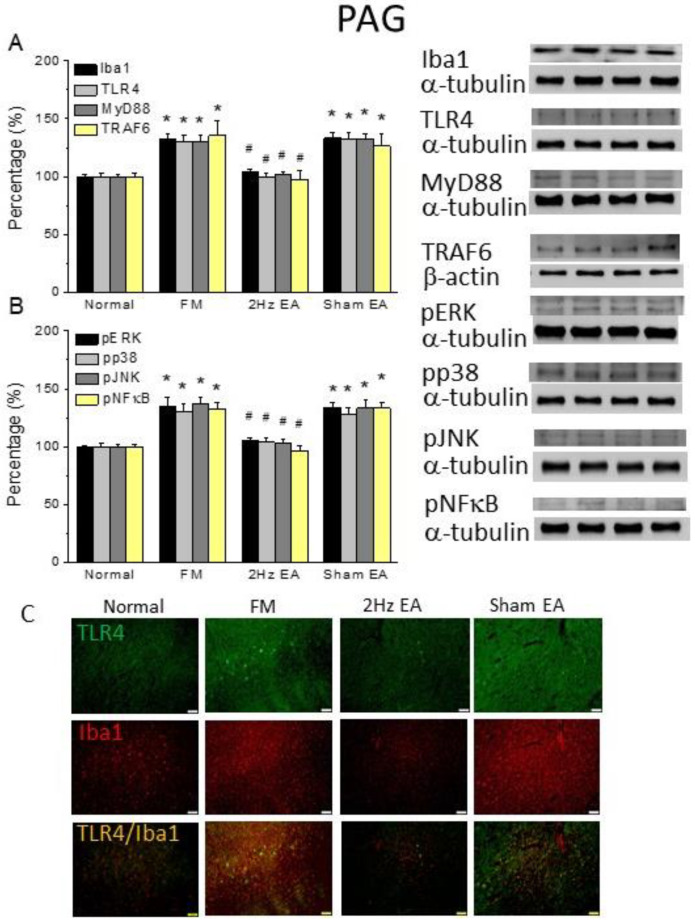
The levels of TLR4 and related molecules in the mice PAG. The Western blot bands have four lanes of protein expression in the order of Normal, FM, 2Hz EA, and sham EA groups. There are significant increases in (**A**) Iba1, TLR4, MyD88, and TRAF6 and (**B**) pERK, pp38, pJNK, and pNFκB protein levels in the FM group. * indicates statistical significance when compared with the normal group. # indicates statistical significance when compared to the FM group. *n* = 6 in all groups. (**C**) Immunofluorescence staining of TLR4, Iba1, and double-staining protein expression in the mice PAG. TLR4, Iba1, and TLR4/Iba1 double-staining, immuno-positive (green, red, or yellow) signals in the mice PAG region. Scale bar means 100 μm. *n* = 4 in all groups.

Western blotting served to explore the Iba1–TLR4 signaling pathway in the mouse hypothalamus and PAG. Iba1 and TLR4 expression was detected in normal mice; in FM mouse hypothalamus, their expression levels increased (Figure 2A, 130.01% ± 5.18% and 127.46% ± 3.08%, black and light gray column, * *p* ˂ 0.05, F(3, 20) = 20.27 and F(3, 20) = 22.88, *n* = 6, respectively). EA at 2 Hz reliably reduced Iba1 and TLR4 overexpression (Figure 2A, 99.53% ± 3.31% and 101.34% ± 2.3%, black and light gray column, # *p* ˂ 0.05, *n* = 6) but not sham EA (Figure 2A, 136.81% ± 5.75% and 126.34% ± 5.04%, black and light gray column, # *p* ˂ 0.05, *n* = 6). Furthermore, we measured MyD88 and TRAF6 levels as TLR4-associated downstream proteins and found that both increased in the FM group (Figure 2A, 127.79% ± 3.69% and 130.96% ± 3.43%, gray and yellow column, * *p* ˂ 0.05, F(3, 20) = 20.26 and F(3, 20) = 18.41, *n* = 6, respectively) and were decreased by EA treatment (Figure 2A, 104.47% ± 2.85% and 100.97% ± 2.33%, gray and yellow column, * *p* ˂ 0.05, *n* = 6) but not sham EA (Figure 2A, 125.15% ± 3.51% and 135.39% ± 7.36%, gray and yellow column, # *p* ˂ 0.05, *n* = 6). Furthermore, we verified that all downstream molecules, including pERK, pp38, and pJNK, were potentiated in the FM mouse hypothalamus (Figure 2B, pERK: 129.73% ± 7.33%, pp38: 138.15% ± 12.42%, and pJNK: 136.69% ± 5.35%, * *p* ˂ 0.05, F(3, 20) = 11.08, F(3, 20) = 10.02, and F(3, 20) = 23.17, *n* = 6, respectively). These increased levels were further reversed in EA mice, but not in sham EA mice (Figure 2B, # *p* ˂ 0.05, *n* = 6). Moreover, we measured the levels of the transcription factor pNFκB in the mouse hypothalamus. We observed that pNFκB increased in both with FM (Figure 2B, 130.88% ± 7.48%, * *p* ˂ 0.05, F(3, 20) = 13.31, *n* = 6) and was reversed by EA but not sham EA (Figure 2B, 99.64% ± 2.14% and 129.32% ± 5.48%, # *p* ˂ 0.05, *n* = 6). Similar results were observed in the mouse PAG (Figure 3A,B, * *p* ˂ 0.05, *n* = 6). These results indicate the contribution of inflammatory factors and TLR4 pathways to the FM phenotype. Moreover, these changes in TRPV1 and related molecules were reversed by EA treatment. We used immunostaining for TLR4 localization on the hypothalamus and PAG, a brain area involved in pain modulation. We observed very low levels of TLR4 in normal mice and increased levels in the FM mouse hypothalamus and PAG (Figure 2 and Figure 3C, green color, *n* = 4). EA, but not sham EA, reliably alleviated this overexpression found in the CSP model (Figure 2 and Figure 3C, green color, *n* = 4). Furthermore, a similar tendency showed increased Iba1, which was further attenuated in the EA group but not in the sham group (Figure 2 and Figure 3C, red color, *n* = 4). Additionally, TLR4 and Iba1 colocalization was detected in the mouse hypothalamus and PAG (Figure 2 and Figure 3C, yellow color, *n* = 4). This pattern of localization was further abrogated by EA but not sham treatment.

### 3.4. TLR4 Signaling in the Cold-Stress-Induced Pain Mouse Cerebellum Was Attenuated by Electroacupuncture but Not Sham Electroacupuncture

**Figure 4:**
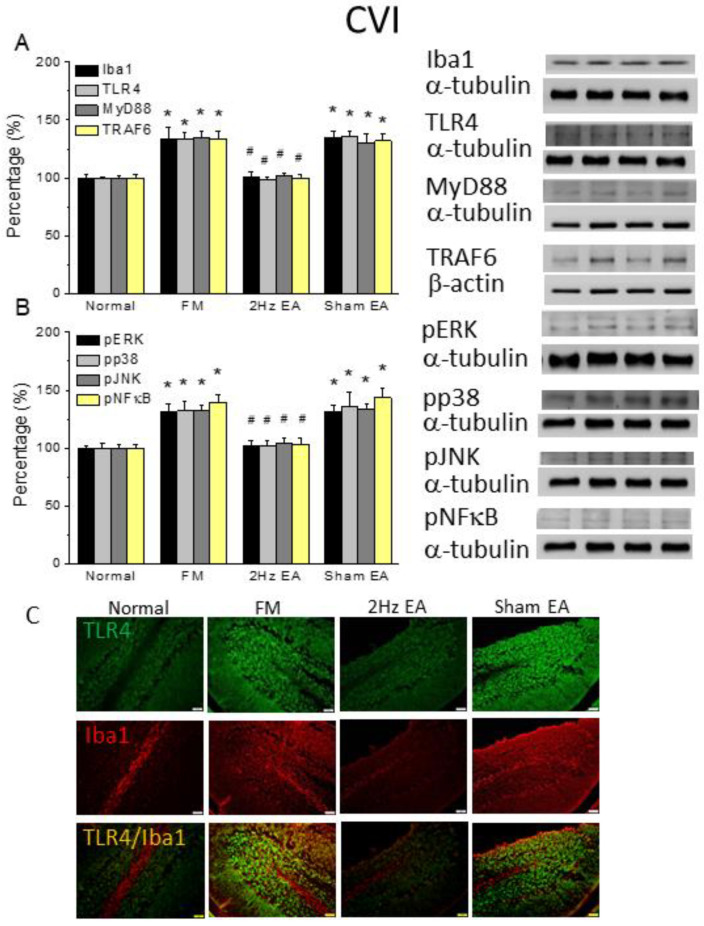
The levels of TLR4 and related molecules in the mice CVI. The Western blot bands have four lanes of protein expression in the order of Normal, FM, 2Hz EA, and sham EA groups. There are significant increases in (**A**) Iba1, TLR4, MyD88, and TRAF6, (**B**) pERK, pp38, pJNK, and pNFκB protein levels in the FM group. * indicates statistical significance when compared with the normal group. # indicates statistical significance when compared to the FM group. *n* = 6 in all groups. (**C**) Immunofluorescence staining of TLR4, Iba1, and double-staining protein expression in the mice CVI. TLR4, Iba1, and TLR4/Iba1 double-staining, immuno-positive (green, red or yellow) signals in the mice CVI region. Scale bar means 100 μm. *n* = 4 in all groups.

**Figure 5:**
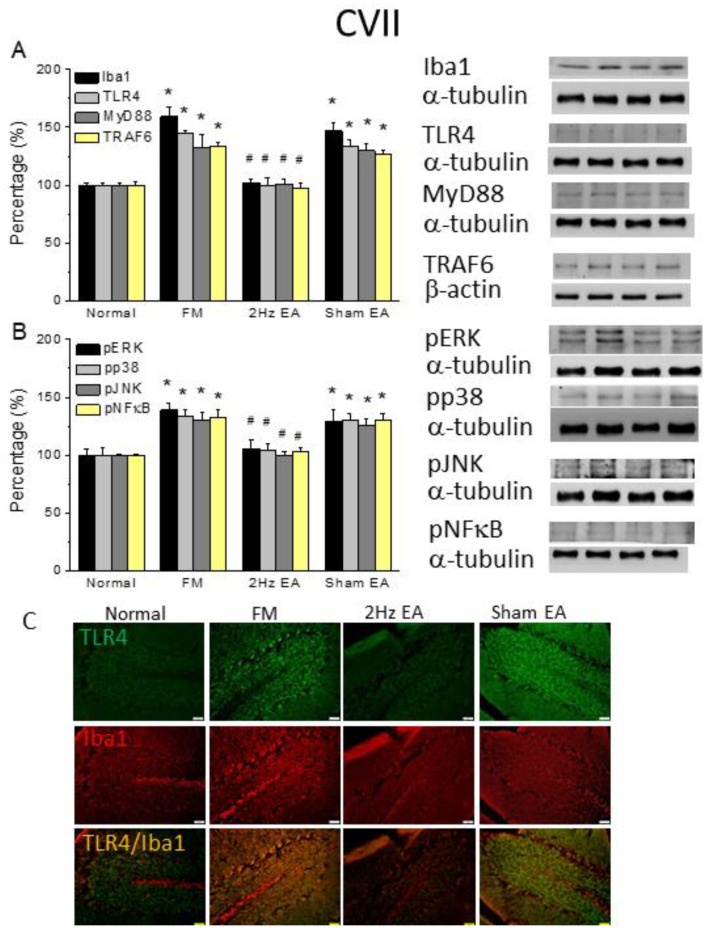
The levels of TLR4 and related molecules in the mice CVII. The Western blot bands have four lanes of protein expression in the order of Normal, FM, 2 Hz EA, and sham EA groups. There are significant increases in (**A**) Iba1, TLR4, MyD88, and TRAF6, (**B**) pERK, pp38, pJNK, and pNFκB protein levels in FM group. * indicates statistical significance when compared with the normal group. # indicates statistical significance when compared to the FM group. *n* = 6 in all groups. (**C**) Immunofluorescence staining of TLR4, Iba1, and double-staining protein expression in the mice CVII. TLR4, Iba1, and TLR4/Iba1 double-staining, immuno-positive (green, red, or yellow) signals in the mice CVII region. Scale bar means 100 μm. *n* = 4 in all groups.

Because the cerebellum plays a critical role in FM pain, we evaluated Iba1 and TLR4 signaling pathways in the mouse cerebellum after FM. Our data showed that Iba1 and TLR4 expression significantly increased after FM induction in the cerebellum lobule VI (Figure 4A, 133.98% ± 9.64% and 133.47% ± 5.73%, * *p* ˂ 0.05, F(3, 20) = 15.46 and F(3, 20) = 30.24, *n* = 6). EA significantly decreased Iba1 and TLR4 increased (Figure 4A, 100.25% ± 4.41% and 98.96% ± 1.75%, # *p* ˂ 0.05, *n* = 6), but this was not the case in sham mice (Figure 4A, 135.02% ± 5.49% and 135.89% ± 4.18%, * *p* ˂ 0.05, *n* = 6). MyD88 and TRAF6 protein levels in the CVI served to characterize TLR4 downstream effectors. Similar to TLR4, MyD88 and TRAF6 levels simultaneously increased after FM induction (Figure 4A, 134.95% ± 5.67% and 133.33% ± 6.77%, * *p* ˂ 0.05, F(3, 20) = 21.47 and F(3, 20) = 20.16, *n* = 6). EA caused a significant decrease in these levels (Figure 4A, 102.26% ± 2.11% and 99.86% ± 3.12%, # *p* ˂ 0.05, *n* = 6) compared to sham EA (Figure 4A, 130.49% ± 7.36% and 131.84% ± 6.71%, * *p* ˂ 0.05, *n* = 6). Increased pERK, pp38, and pJNK were also detected in FM mice (Figure 4B, 131.52% ± 6.4%, 131.88% ± 8.31%, and 139.61% ± 5.95%, * *p* ˂ 0.05, F(3, 20) = 17.43, F(3, 20) = 7.93, and F(3, 20) = 25.03, *n* = 6, respectively). However, their levels significantly decreased in mice treated with EA (Figure 4B, 102.01% ± 4.99%, 101.78% ± 5.19%, and 104.78% ± 4.26%, # *p* ˂ 0.05, *n* = 6) but not with sham EA (Figure 4B, 131.43% ± 6.04%, 135.7% ± 12.52%, and 134.14% ± 4.59%, * *p* ˂ 0.05, *n* = 6). With respect to pNFκB, the protein levels of pNFκB increased in FM mice, an increase that was further reversed with EA treatment (Figure 4B, 139.61% ± 5.95%, and 103.69% ± 5.04%, * *p* ˂ 0.05, F(3, 20) = 22.96, *n* = 6). The aforementioned tendency, as a TLR4 signaling pathway, was also obtained in CVII, suggesting its crucial role in FM and EA treatment effects (Figure 5, * *p* ˂ 0.05, *n* = 6).

Furthermore, we tried to identify whether TLR4 and Iba1 levels were altered and distributed in the mouse cerebellum, a brain area involved in patients with FM. Our data indicated that TLR4 was distributed in the CVI and VII in normal mice. TLR4 levels further increased after FM induction in the mouse CVI and VII (Figure 4 and Figure 5C, green color, *n* = 4) and were then reversed by EA, but not sham EA (Figure 4 and Figure 5C, green, *n* = 4). Similarly, Iba1 increased with FM and was attenuated in EA, but not in sham EA (Figure 4 and Figure 5C, red color, *n* = 4). Moreover, double-staining of TLR4 and Iba1 were observed in the mice CVI and VII (Figure 4 and Figure 5C, yellow color, *n* = 4); a pattern abrogated by EA but not sham treatment.

### 3.5. Activation of TLR4 by LPS Injection Mimics CSP and Further Reversed by TLR4 Antagonist TIRAP

**Figure 6:**
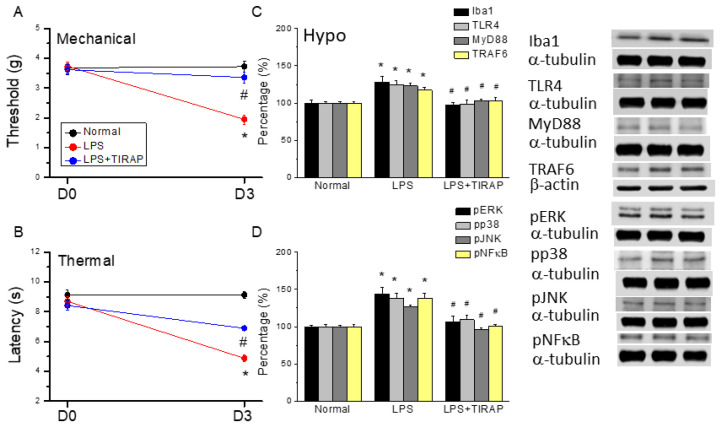
Mechanical, thermal hyperalgesia, and Western blot analysis in normal, LPS, and TIRAP mice. (**A**) Mechanical threshold from the von Frey tests. (**B**) Thermal latency from the Hargreaves’ test. (**C**) Iba1, TLR4, MyD88, and TRAF6, (**D**) pERK, pp38, pJNK, and pNFκB protein levels in all groups. * indicates statistical significance when compared with the normal group. # indicates statistical significance when compared to the LPS group. *n* = 6 in all groups.

Recent articles showed that alterations in higher brain areas are essential for developing chronic pain [12,28]. Therefore, we hypothesized that activation of central TLR4 is involved in the central sensitization of chronic pain. Intracerebral ventricle infusion of LPS, which is an agonist of TLR4, significantly induced mechanical hyperalgesia (Figure 6A, 1.95 ± 0.16 g, * *p* ˂ 0.05, *n* = 6). Furthermore, co-administration of LPS and toll-interleukin 1 receptor domain-containing adapter protein (TIRAP) reliably reversed this phenomenon (Figure 6A, 3.36 ± 0.19 g, * *p* ˂ 0.05, *n* = 6). A similar tendency was also observed in thermal hyperalgesia (Figure 6B, * *p* ˂ 0.05, *n* = 6). By using Western blot, we indicated that Iba1 and TLR4 level were significantly augmented after LPS injection in the mouse hypothalamus (Figure 6C, 128.43% ± 7.17% and 124.4% ± 5.58%, * *p* ˂ 0.05, *n* = 6). TIRAP meaningfully diminished Iba1 and TLR4 rises (Figure 6C, 97.57% ± 3.43% and 98.37% ± 5.76%, # *p* ˂ 0.05, *n* = 6). MyD88 and TRAF6 levels served to describe TLR4 downstream effectors. Similarly, MyD88 and TRAF6 proteins were concurrently augmented after FM induction (Figure 6C, 122.67% ± 3.79% and 117.29% ± 3.77%, * *p* ˂ 0.05, *n* = 6). TIRAP produced a significant reduction in these levels (Figure 6C, 103.04% ± 2.58% and 103.41% ± 3.78%, # *p* ˂ 0.05, *n* = 6). Amplified pERK, pp38, and pJNK were also identified in the LPS group. However, their expressions dramatically declined in TIRAP-treated mice (Figure 6D, * *p* ˂ 0.05, *n* = 6). The expression of pNFκB was increased in LPS mice and then reversed with TIRAP treatment (Figure 6D, * *p* ˂ 0.05, *n* = 6).

## 4. Discussion

FM is a complicated symptom with chronic pain, fatigue, sleep disturbance, obesity, and depression affecting the whole body. FM is often resistant to opioid drugs or nonsteroidal anti-inflammatory drugs [28]. Additionally, antidepressants SNRI and GABAergic medications (pregabalin) are the most commonly used drugs with several side effects. Accordingly, EA has a beneficial effect and chance to relieve FM. Vas et al. reported that acupuncture is an effective treatment for FM [29]. Langhorst et al. also verified that acupuncture treatment for FM is greater than medicines. Acupuncture combined with medicines and exercise noticeably increases pain thresholds [30]. Neyama and Ueda initiated an ideal FM model named the ICS model, which mimics similar symptoms in patients with FM. Gabapentin had a significant antiallodynic effect, but not in the morphine-treated group. Patients with FM also have characteristic emotional problems, including depression. Our recent publication indicated that mice subjected to the ICS process presented depression symptoms as in the human clinical presentation [12]. Our previous data suggest that acid saline injection-induced chronic pain and depression comorbidity produce changes in the cerebellum lobules VI, VII, and VIII. EA significantly ameliorated these phenomena through action on TRPV1 and related molecular pathways [31].

TLR4 was reported to be expressed in both neuronal and glial cells in the CNS responding to inflammatory mediators. In inflammation or nerve injury, TLR4 was suggested to activate microglia and astrocytes to induce the release of inflammatory cytokines at the spinal cord level to develop inflammatory pain and neuropathic pain [32,33,34]. Microglia have been reported as non-neuronal cells that have TRPV1 receptors, and EA can inhibit the glial TRPV1 receptors to prevent S100B secretion to activate the receptor for advanced glycation end-products on the neuronal membrane. This results in the inhibition of inflammatory processes for pain relief. In our previous publication, we reported that EA can prevent neuronal TRPV1 and inactivate the down-regulating signaling pathways [35]. Our previous results showed that EA triggers endomorphin and adenosine release to attenuate inflammatory pain in the peripheral and central nervous systems of mice [21]. Xu et al. indicated that the blockage of the TLR4 signaling pathway could attenuate chronic constriction-injury-induced neuropathic pain by measuring mechanical withdrawal threshold and thermal withdrawal latency [36]. This study indicated that EA significantly attenuated FM accompanied by decreasing the TLR4/MyD88/NF-κB pathway.

The study results proved that several inflammatory mediators increased in the circulating blood of FM mice and can be attenuated by EA, suggesting an anti-inflammatory effect. In the neuropathic pain model, mechanical or thermal hyperalgesia was abrogated after inhibition of TLR4. Ma and colleagues suggested that TLR4 increased in the neuropathic pain model [37]. Inhibition of inflammatory cytokines and TLR4 was reported to attenuate the pain threshold in neuropathic pain mice [38]. Our data indicated that TLR4 and downstream molecules increased in FM mice and can be further reduced after EA treatment. Furthermore, we found that TLR4 was colocalized with Iba1 and simultaneously increased after FM induction. EA, but not sham EA, significantly reduced these phenomena. A recent article showed that the mRNA and protein levels of TLR4, TNF-α, and IL-1β were simultaneously increased. Additionally, the expression of NF-κB was also found to increase in the spinal dorsal horn [39]. Furthermore, inhibition of TLR4 significantly reversed the hyperalgesia and inflammatory factors, indicating its crucial role in the neuropathic pain model [36]. The current results suggest that EA can attenuate the overexpression of TLR4, TNF-α, IL-1β, and NF-κB in FM mice [39,40]. This study provides evidence of how TLR4 and associated molecules participate in the brain regions of this murine fibromyalgia model. The novel discoveries are relevant for the treatment of fibromyalgia in clinical practice. Future investigations should be conducted through clinical trials.

## 5. Conclusions

**Figure 7:**
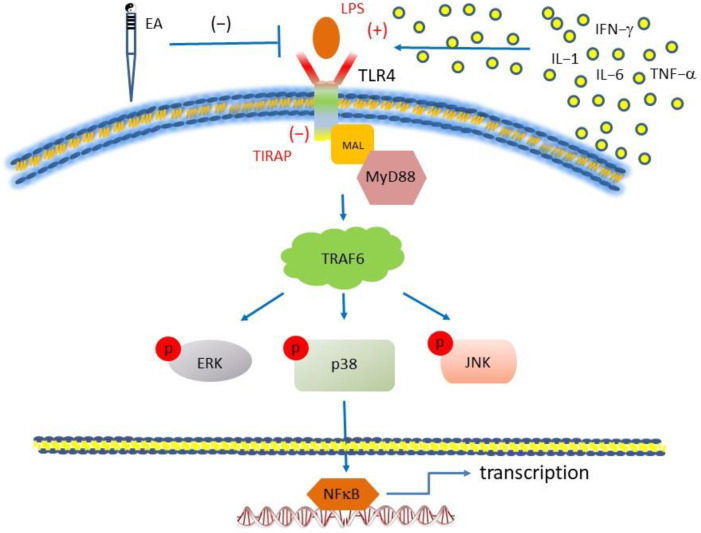
Illustration of mechanisms underlying the EA-mediated analgesic effect on FM pain. Summary illustration displays the importance and mechanisms involving cells and TLR4 in FM pain. EA inhibits the release of inflammatory cytokines from non-neuronal cells or directly inhibits TLR4 on the plasma membrane.

In conclusion, the main outcomes in this study are that FM induction significantly activates pain, inflammation, and central sensitization in a mouse central pain model. Mechanical and thermal hyperalgesia were observed in the FM mice. Inflammation contains increased IL-1β, IL-2, IL-5, IL-6, IL-9, IL-12, IL-17A, TNF-α, IFN-γ, and MCP-1 in the mice plasma. Furthermore, we have confirmed that TLR4 and related molecules increased in the mice’s hypothalamus, PAG, and cerebellum. Particularly, we reported TLR4 is an inflammation detector for FM. Additionally, inflammatory modulators were released to activate TLR4 for pain processing. All signals further activate the aforementioned brain regions for the pain signaling pathway, including MYD88 and TRAF6. EA potently suppressed these complicated molecular pathways in the FM mice brain (Figure 7).

## Data Availability

The data used to support the findings of this study are available from the corresponding author upon request.
